# Metabolic and Behavioural Phenotypes in Nestin-Cre Mice Are Caused by Hypothalamic Expression of Human Growth Hormone

**DOI:** 10.1371/journal.pone.0135502

**Published:** 2015-08-14

**Authors:** Jeroen Declercq, Bas Brouwers, Vincent P. E. G. Pruniau, Pieter Stijnen, Geoffroy de Faudeur, Krizia Tuand, Sandra Meulemans, Lutgarde Serneels, Anica Schraenen, Frans Schuit, John W. M. Creemers

**Affiliations:** 1 Laboratory for Biochemical Neuroendocrinology, Department of Human Genetics, KU Leuven, Leuven 3000, Belgium; 2 Gene Expression Unit, Department of Cellular and Molecular Medicine, KU Leuven, Leuven 3000, Belgium; 3 Laboratory for the Research of Degenerative Diseases, KU Leuven, Leuven 3000, Belgium; Pennington Biomedical Research Center/LSU, UNITED STATES

## Abstract

The Nestin-Cre driver mouse line has mild hypopituitarism, reduced body weight, a metabolic phenotype and reduced anxiety. Although several causes have been suggested, a comprehensive explanation is still lacking. In this study we examined the molecular mechanisms leading to this compound phenotype. Upon generation of the Nestin-Cre mice, the human growth hormone (*hGH*) minigene was inserted downstream of the Cre recombinase to ensure efficient transgene expression. As a result, hGH is expressed in the hypothalamus. This results in the auto/paracrine activation of the GH receptor as demonstrated by the increased phosphorylation of signal transducer and activator of transcription 5 (STAT5) and reduced expression of growth hormone releasing hormone (*Ghrh*). Low Ghrh levels cause hypopituitarism consistent with the observed mouse growth hormone (mGH) deficiency. mGH deficiency caused reduced activation of the GH receptor and hence reduced phosphorylation of STAT5 in the liver. This led to decreased levels of hepatic *Igf-1* mRNA and consequently postnatal growth retardation. Furthermore, genes involved in lipid uptake and synthesis, such as CD36 and very low-density lipoprotein receptor were upregulated, resulting in liver steatosis. In conclusion, this study demonstrates the unexpected expression of hGH in the hypothalamus of Nestin-Cre mice which is able to activate both the GH receptor and the prolactin receptor. Increased hypothalamic GH receptor signaling explains the observed hypopituitarism, reduced growth and metabolic phenotype of Nestin-Cre mice. Activation of either receptor is consistent with reduced anxiety.

## Introduction

Mouse models have proven to be versatile tools in biomedical research. Numerous publications have used knockout and transgenic mouse models. The discovery of the Cre-Lox recombinase in bacteriophage P1 and its application in conditional knockout models has advanced the field enormously [1]. This system allows the excision of a DNA fragment that is flanked by two loxP sites (referred to as floxed), using the enzyme Cre-recombinase. Since this discovery, numerous floxed mouse models have been generated. These mouse lines can be crossed with Cre-driver lines that express the Cre-recombinase under the control of a tissue-specific or inducible promoter, allowing inactivation of genes in a temporo-spacial manner. However, several problems are associated with this technology. The majority of Cre-driver lines are generated by pronuclear microinjection, in which the genomic integration site of the Cre-recombinase transgene is not controlled [2]. Therefore, it can potentially disrupt the expression of endogenous genes. Furthermore, it can result in ectopic, low, or even mosaic expression of Cre-recombinase [3]. Cre expression can also mediate genomic alterations which are independent of LoxP sites [4] and induce apoptosis or decrease proliferation in cell lines [5]. *In vivo* it has been shown that this can lead to brain damage [6, 7]. For all those reasons it is essential to include the Cre-driver lines as control mice in Cre-LoxP studies. Nevertheless, this control is often not included in those studies, making it difficult to fully interpret the results. In recent years, physiological problems have been reported for several Cre-driver lines [8, 9], including Nestin-Cre mice [10–12]. The Nestin-Cre mouse model is frequently used to drive deletions to the central nervous system (CNS) and the peripheral nervous system (PNS) [13]. Nestin is an intermediate filament protein that is highly expressed in neuronal progenitor cells. It has been reported that the expression pattern of the Cre transgene is not specific to the CNS and PNS, but that the transgene is also expressed in several other tissues, like the pancreas and the kidneys [14, 15]. Furthermore, the Nestin-Cre mice have hypopituitarism resulting in significantly decreased levels of growth hormone and decreased body weight [10]. Moreover, Nestin-Cre mice show reduced contextual- and cued-conditioned fear [11]. Finally, Nestin-Cre mice have higher adiposity and circulating leptin levels and they are less tolerant to glucose challenge and more sensitive to insulin administration [16].

Although it is clear that the Nestin-Cre mice have many physiological problems, it is still unknown why this is the case. It has been suggested that this might be due to the integration site of the transgene [17] or the toxic effect of Cre in neurons [11]. However, in this study we have investigated another mechanism potentially underlying this artifact, caused by the construct used to generate the Nestin-Cre mice. Upon generation of the Nestin-Cre mice, the human growth hormone (hGH) minigene, including the entire coding region, introns and polyadenylation signal was inserted downstream of the Cre recombinase [13]. This strategy is used for the generation of several transgenic mouse models, since it was shown that intronic sequences and a polyadenylation signal are essential to achieve efficient expression of the *Cre* transgene [18, 19]. hGH can activate both the mouse GH receptor and prolactin receptor (PRLR) [20]. We have examined the expression of hGH in the hypothalamus, pituitary gland, and liver of Nestin-Cre mice and its downstream signaling.

## Materials and Methods

### Mice breeding

Heterozygous Nestin-Cre mice (C57Bl/6J-Tg(Nes-cre)1Kln) backcrossed at least 10 times to a C57BL/6J background were housed in standard cages (wood-shaving bedding) on a 12-hour day/night cycle (lights on at 8am) and were fed a standard rodent chow. The total body weight was measured at three months. Mice were sacrificed by cervical dislocation. All experiments with laboratory animals were approved by the ethical research committee for animal welfare at the KU Leuven in accordance with the declaration of Helsinki (KU Leuven project number 036/2015).

### Reverse transcription quantitative PCR (RT-qPCR)

Total RNA was isolated from the pituitary gland, the hypothalamus and the liver of 3-month-old male Nestin-Cre mice using the Nucleospin RNA midi kit (Macherey Nagel, Düren, Germany) according to the manufacturer’s protocol. First strand cDNA was synthesized using iScript cDNA synthesis kit (Bio-Rad, Hercules, CA). Primers sequences are listed in Table 1. RT-qPCR was performed in triplicate with MyIQ Single Color Real-Time PCR Detection System (Bio-Rad) using SYBR Green. Samples were normalized to glyceraldehyde 3-phosphate dehydrogenase (*Gapdh*). Data were analysed using the Livak method [21]. *Cish* expression was quantified using primers and a Taqman probe (Table 1) on a Rotorgene (Corbett Research). To detect the *Cre-hGH* fusion transcript, PCR was performed using MyTaq polymerase (Bioline, London, UK), with a forward primer annealing to the 3’ end of Cre and a reverse primer annealing to the 5^th^ exon of hGH.

**Table 1:** Primer sequences used for transcript detection.

Gene	Sequence
*Gapdh*	Forward: 5’ CCCCAATGTGTCCGTCGTG 3’ Reverse: 5’ GCCTGCTTCACCACCTTCT 3’
*hGH*	Forward: 5’ CCAGGAGTTTGAAGAAGCCT 3’ Reverse: 5’ ggaggtcatagacgttgctgt 3’
*Cre-hGH (QPCR)*	Forward: 5’ CTATATCCGTAACCTGGATAGTG 3’ Reverse: 5’ AGGCTTCTTCAAACTCCTGG 3’
*Cre-hGH*	Forward: 5’ CTATATCCGTAACCTGGATAGTG 3’
*(PCR)*	Reverse: 5’ CTTGAAGATCTGCCCAGTCC 3’
*Ghrh*	Forward: 5’ gcagaacctcaatcggagag 3’
	Reverse: 5’ tggtgaggatgaggatcaca 3’
*Igf1*	Forward: 5’ TTTTACTTCAACAAGCCCACAGG 3’
	Reverse: 5’ AGGTGCCCTCCGAATGC 3’
*CD36*	Forward: 5’ TGCATGAATTAGAACCGGGCCA 3’
	Reverse: 5’ AGCTCCAGCAATGAGCCCAC 3’
*Cish*	Forward: 5’ AAGGTGCTAGACCCTGA 3’
	Probe: 5’ (6-FAM)ATAGCCAAGACGTTCTCCTACCTTCGGGAAT(TAMRA) 3’
	Reverse: 5’ CTCGCTGGCTGTAATAGAA 3’
*Vldlr*	Forward: 5’ GAGCCCCTGAAGGAATGCC 3’
	Reverse: 5’ CCTATAACTAGGTCTTTGCAGAT 3’

### hGH ELISA

Mouse hypothalamus, pituitary and liver samples were isolated and lysed in 1x RIPA buffer supplemented with complete protease inhibitors (Roche). The hGH content in these tissues was measured and calculated using a HGH human direct ELISA kit (Invitrogen, Paisley, UK) according to the manufacturer’s protocol.

### Western blot analysis

Livers were dissected and snap frozen in liquid nitrogen. Snap frozen tissues were homogenized in Cell Lysis Buffer (Cell Signaling Technology) supplemented with complete protease inhibitors (Roche) and phosphoSTOP (Roche). Phosphorylation of signal transducer and activator of transcription (STAT5) was analyzed by western blot analysis using standard procedures. Rabbit anti-mouse STAT5 (1/1000, Cell Signaling Technology) and rabbit anti-mouse phospho-STAT5 (Tyr694) (1/1000, Cell Signaling Technology) antibodies diluted in PBS with 5% (w/v) nonfat dry milk and 0.2% (v/v) Triton X100 were used as primary antibodies. Detection of proteins was carried out with the ECL method using the Western Lightning enhanced luminol-based chemiluminescence HRP substrate (Perkin Elmer). The phospho-STAT5/STAT5 ratio in the hypothalamus was determined using ImageJ software (National Institutes of Health).

### Statistical analysis

Unpaired student t-tests were performed for the statistical analysis. Data are represented as mean ± SD. * p<0.05, **p<0.01, ***p<0.001.

## Results

**Fig 1:**
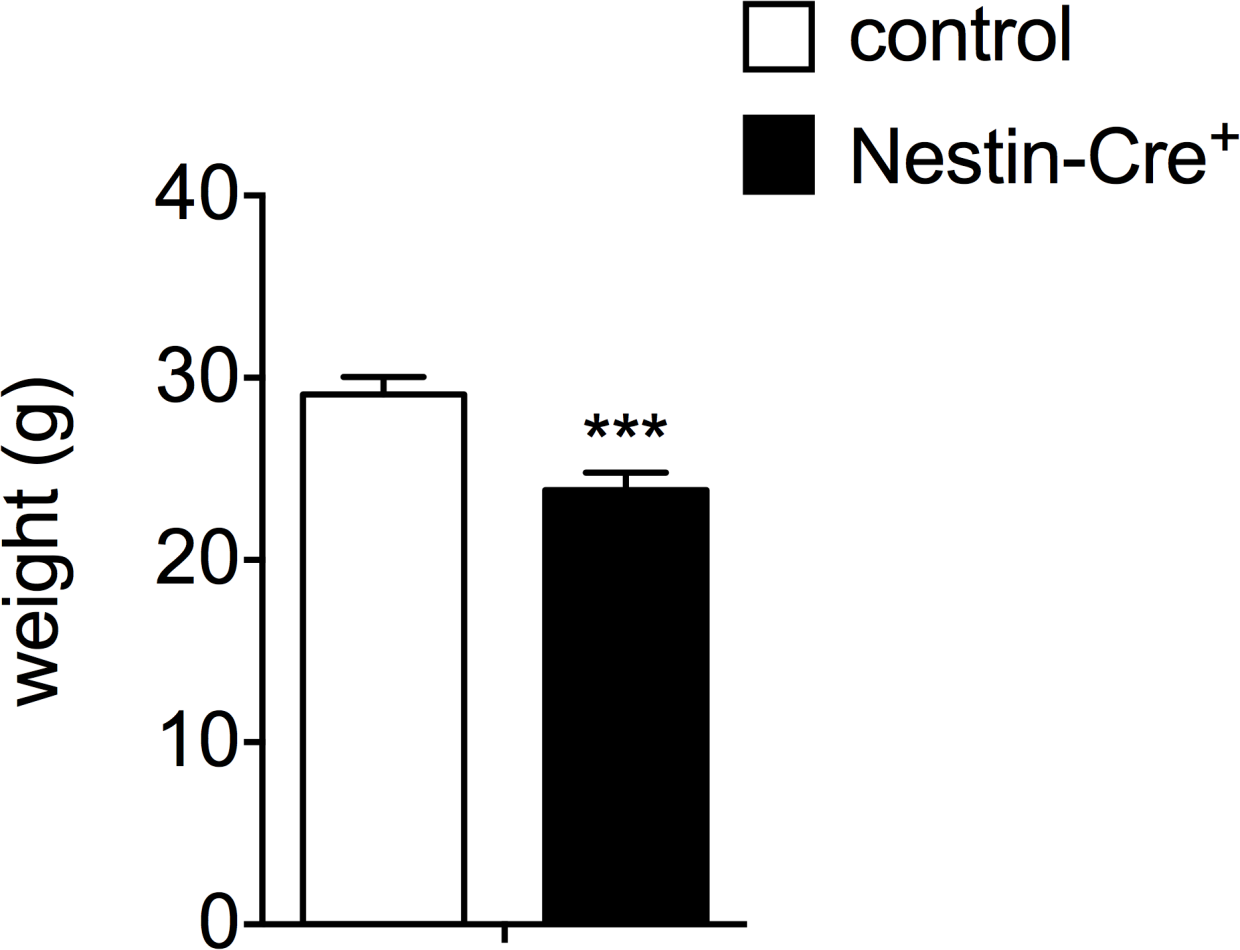
The total body weight of Nestin-Cre mice is reduced. The total body weight of 3-month-old Nestin-Cre and wild type male mice (n = 4–5). Data are represented as mean ± SD. ***p<0.001.

**Fig 2:**
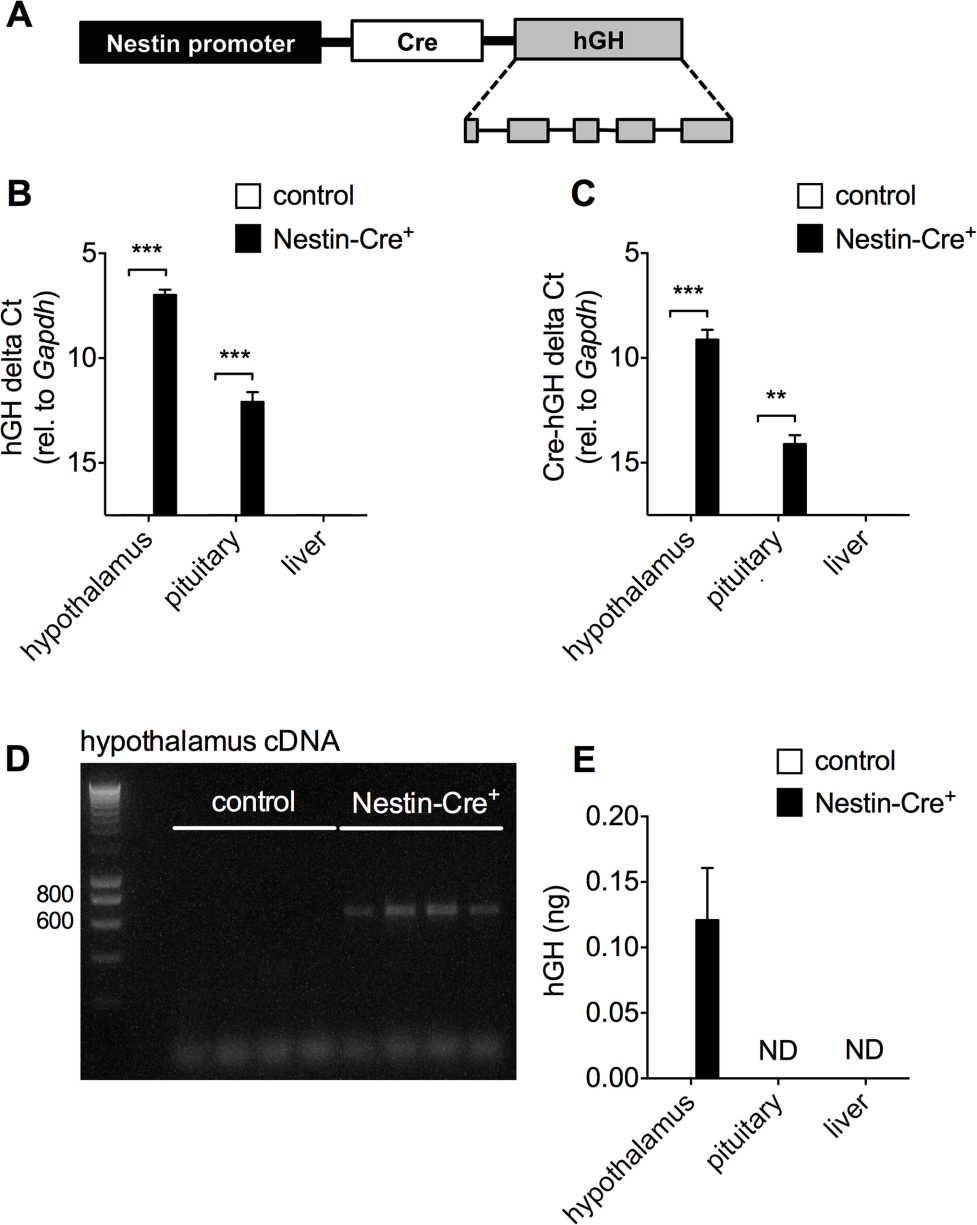
*hGH* is expressed in the hypothalamus and to a lesser extent in the pituitary gland of Nestin-Cre mice. A) Schematic representation of the Cre-hGH transgene based on Tronche et al. [13] B) The expression of *hGH* was investigated by RT-qPCR in the hypothalamus, the pituitary gland and the liver of 3-month-old male Nestin-Cre mice and control littermates (n = 4–5). C) Detection of a bicistronic Cre-hGH transcript by RT-qPCR, using a forward primer annealing to Cre and a reverse primer annealing to hGH. (n = 4–5) D) PCR was performed on hypothalamus cDNA from control and Nestin-Cre male mice (n = 4 per group), using primers in Cre and the last exon of hGH. E) hGH ELISA was performed on hypothalamus, pituitary and liver lysates from Nestin-Cre and control littermates (n = 6 for both groups). ND, not detected. Data are shown as mean ± SD, * p<0.05, **p<0.01, ***p<0.001.

The three-month-old male Nestin-Cre mice in a C57BL/6J background used in this study, showed a significant decrease in the total body weight as compared to control littermates (29.10 ± 0.96 g compared to 23.85 ± 0.94 g for controls, Fig 1). In an attempt to explain this aspect of the compound phenotype of Nestin-Cre mice, the transgenic construct used for the pronuclear microinjection was scrutinized. The original report of this mouse model showed that the hGH minigene was inserted downstream of the Cre recombinase, to achieve a higher expression level of the transgene (Fig 2A) [13]. Expression of *hGH* in the hypothalamus and to a much lower extent in the pituitary gland, but not in the liver was demonstrated by RT-qPCR (Fig 2B). Similar RT-qPCR signals were found when a forward primer annealing to the Cre fragment and a reverse primer annealing to the junction between exon 2 and 3 of the hGH minigene were used, indicating a single mRNA (Fig 2C). To provide further evidence that the full open reading of hGH was included, PCR was performed on cDNA from hypothalamus using one primer in *Cre* and the other in exon 5 of *hGH* (Fig 2D). A band of ~650 bp was detected in all Nestin-Cre animals, consistent with the last 64 bp region of the *Cre* open reading frame, a short bridge region between Cre and hGH, and the 535 bp fragment of the open reading frame of *hGH*. Since no band was detected in littermate controls, cross-reactivity with *mGH* can be excluded. To measure possible translation of the open reading frame of *hGH* from this mRNA, a hGH ELISA was performed on hypothalamus, pituitary and liver samples from Nestin-Cre mice versus littermate controls. hGH protein levels were detected only in the hypothalamus of Nestin-Cre animals (0.12 ± 0.04 ng/hypothalamus), and not in pituitary or liver (Fig 2E). It is unlikely that the antibody would detect mouse GH, as no appreciable signal was obtained in samples from non-transgenic control mice; therefore, we consider these assays to reflect the production of human GH encoded by the transgene. Given the lower *hGH* mRNA signal in pituitary compared to hypothalamus, it is possible that hGH is present in this tissue, but below the detection limit of the ELISA.

**Fig 3:**
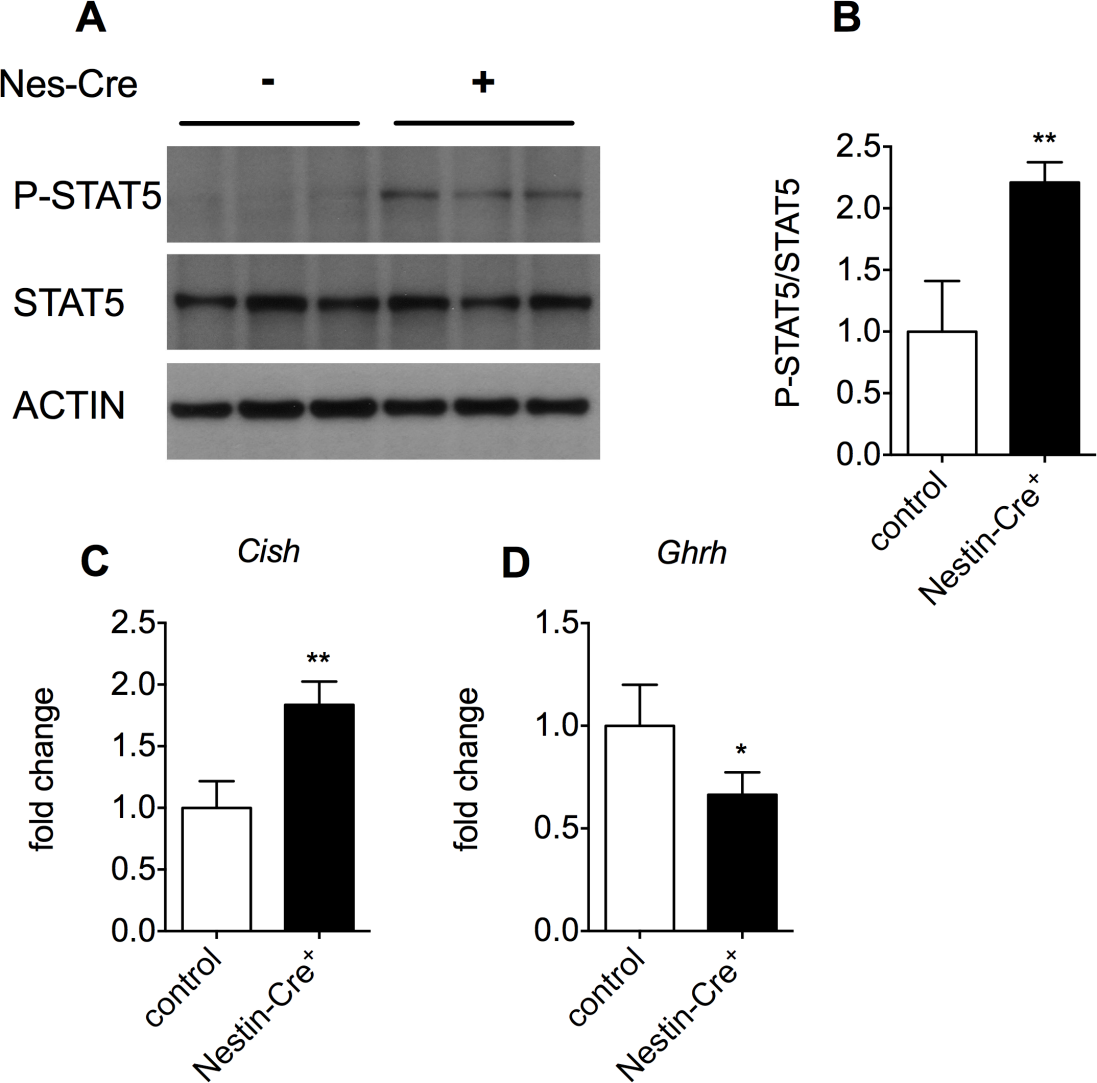
Hypothalamic hGH expression increases STAT5 phosphorylation, induces *Cish* expression and leads to a reduction in the expression of *Ghrh*. A) Immunoblotting for STAT5 and phospho-STAT5 on hypothalamus lysates from Nestin-Cre versus control male mice. Three independent samples are shown per genotype. Actin was used as a loading control. B) Quantification of the phospho-STAT5/STAT5 ratio, **p<0.01. C) Hypothalamic *Cish* expression as quantified by RT-qPCR, n = 4–5, **p<0.01. D) The expression of *Ghrh* was investigated by RT-qPCR in the hypothalamus of 3-month-old male Nestin-Cre mice and control littermates, n = 4, *p<0.05.

Expression of hGH resulted in increased phosphorylation of hypothalamic STAT5, (2.20 ± 0.16-fold, Fig 3A and 3B). Phospho-STAT5 is a downstream signaling component of the GH receptor and the prolactin receptor (PRLR), both of which can be activated by hGH and are present in the hypothalamus [20, 22–24]. Downstream of STAT5, cytokine inducible SH2-containing protein (*Cish*) expression was increased in the hypothalamus (1.84 ± 0.20-fold, Fig 3C). In addition, it has previously been described that targeted expression of hGH in the hypothalamus reduces the expression of hypothalamic GH releasing hormone (*Ghrh*), leading to GH deficiency (GHD) [25]. Consistent with this study, hGH expression in the hypothalamus of 3-month-old Nestin-Cre mice results in a 33% decrease of *Ghrh* expression as compared to wild type littermates (Fig 3D). This is most likely the cause of the lower mouse growth hormone (mGH) levels seen in these animals [10].

**Fig 4:**
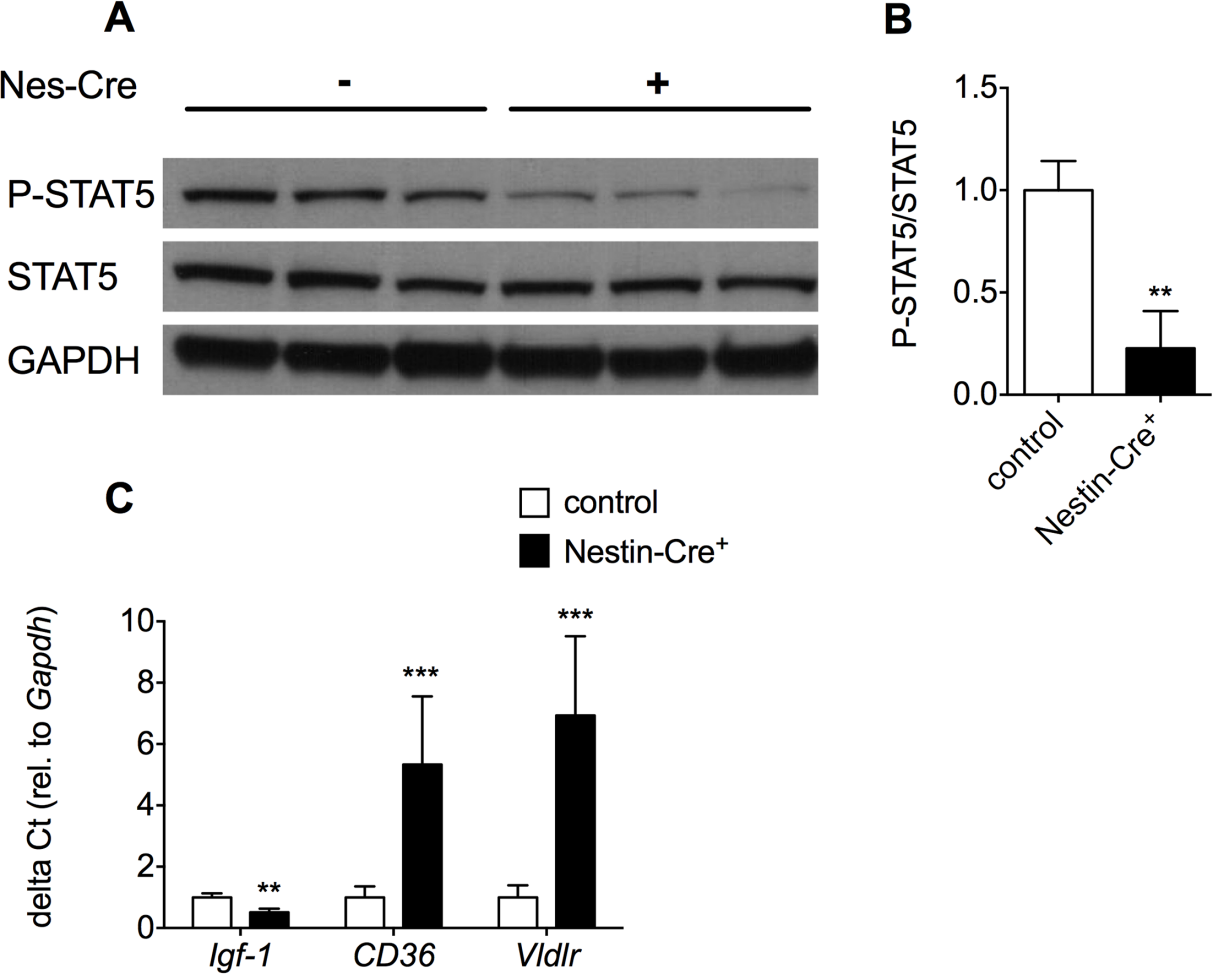
GHD in Nestin-Cre mice leads to a decrease in STAT5 phosphorylation, lower expression of *Igf1* and an increase in the expression of *CD36* and *Vldlr* in the liver. A) Western blot analysis was performed for STAT5 and phospho-STAT5 on liver lysates from 1-month-old male Nestin-Cre mice and control littermates. B) Quantification of the phospho-STAT5/STAT5 ratio, **p<0.01. C) The expression of *Igf1*, *CD36* and *VLDLR* was investigated by RT-qPCR in the liver of 3-month-old male Nestin-Cre mice and control littermates (n = 3–4). Data are represented as mean ± SD. * p<0.05, **p<0.01, ***p<0.001.

GHD in Nestin-Cre mice was also apparent from the decreased phosphorylation of STAT5 in the liver (Fig 4A). Upon binding of mGH to the dimeric GHR in the liver, the GHR undergoes conformational changes that induce transphosphorylation of Janus-family tyrosine kinase 2 (JAK2) and initiation of GHR signaling [26]. Subsequently, JAK2 phosphorylates multiple tyrosines on the intracellular domain of the GHR, which is essential for STAT5 phosphorylation. Therefore, decreased release of mGH from the pituitary gland in Nestin-Cre mice leads to reduced phosphorylation and hence reduced activation of STAT5 in the liver of Nestin-Cre mice. As a consequence, the expression of *Igf1* in the liver is significantly decreased to 51% of the wildtype levels as shown by RT-qPCR (Fig 4B). Decreased IGF1 levels can, at least partially, explain the reduced body weight of Nestin-Cre mice. The metabolic phenotype observed in Nestin-Cre mice can also be explained by GHD [16], since GHD has been associated with abnormal liver lipid profiles and liver steatosis [27]. For instance, mice in which *Stat5* is genetically ablated in the liver show increased lipid uptake and liver steatosis [28]. This can be explained by elevated levels of CD36, which leads to an increased uptake of free fatty acids. Furthermore, liver-specific *Stat5* deficient mice also have increased levels of the very-low-density-lipoprotein receptor (VLDLR), which is associated with liver steatosis. Likewise, in Nestin-Cre mice, the expression of *CD36* and *Vldlr* were increased 5.3 and 6.9 times, respectively (Fig 4B).

## Discussion

In this study we have shown that the previously reported physiological abnormalities associated with the Nestin-Cre driver line may, at least partially, be explained by the ectopic expression of hGH in hypothalamus. As a consequence of hGH expression in hypothalamus, *GHRH* expression is reduced, most likely through auto/paracrine activation of the GH receptor-mediated negative feedback loop [25]. Reduced levels of GHRH cause hypopituitarism, as also observed in for instance GHRH^-/-^ and PC1/3^-/-^ mice [29, 30]. This leads to lower levels of *mGH* in somatotrophs and impaired downstream signaling of mGH in liver through the GH receptor as evidenced by reduced STAT5 phosphorylation. Reduced GH signaling in liver caused reduced *Igf-1* expression and impaired growth, as well as increased levels of *CD36* and *Vldlr*, which is consistent with the reported steatosis and altered lipid profile in the liver of Nestin-Cre mice.

It has been proposed that these abnormalities in Nestin-Cre mice might be explained by the integration site of the transgene, since the Nestin-Cre mice are generated by pronuclear injection. Although we cannot formally exclude this hypothesis, our observations make this explanation unlikely. Similar phenotypic abnormalities as described for Nestin-Cre mice have also been observed in AlfP-Cre mice [9]. AlfP-Cre mice also show GHD and consequently a decreased body weight and a metabolic phenotype. Strikingly, those mice are generated using a similar approach as for the Nestin-Cre mice. They are generated by pronuclear injection of a construct that contains the hGH minigene downstream of the Cre recombinase. Almost certainly, the integration site of the transgene in both mouse models will be different, since the construct was randomly inserted into the genome. Nevertheless, AlfpCre mice express hGH in the hypothalamus and pituitary gland. In these mouse models, it is most likely that hGH is produced from mRNA expression driven by the promoter that was inserted upstream of the Cre coding sequence. Although internal ribosome entry sites are rare in mammalian mRNAs, it has recently been shown for several pancreatic Cre driver lines that both Cre and hGH were translated as independent proteins from the same mRNA [31]. Similarly, the *hGH* mRNA expression profile corresponded to the *Cre* expression pattern in the Nestin-Cre mouse line, showing low mRNA expression levels in pituitary (Fig 2B and [10]). On the other hand, hGH protein is not always detected in all tissues that express the *Cre-hGH* mRNA. In this regard, AlfpCre mice express high levels of the transgenic mRNA in liver, but hGH protein was not observed in this tissue [9]. The other suggested explanation, toxicity of Cre protein expression *per se*, as was previously observed in neuronal progenitor cells [6], cannot be formally excluded, but seems unlikely in light of the current data.

It has previously been described that expression of hGH in the hypothalamus results in GHD [25, 32], explaining the growth retardation and the metabolic phenotype observed in Nestin-Cre mice. A highly similar phenotype was found in a transgenic mouse model in which a hGH genomic fragment was serendipitously inserted into the opposite strand of the *Nbea* gene [33]. These mice also express hGH in hypothalamus and pituitary and have a dwarf phenotype. It is unlikely that hGH expression in this mouse model is able to compensate for GHD, since it was shown that hGH levels were three orders of magnitude lower than endogenous mGH [33]. For these reasons we believe that the construct used to generated Nestin-Cre mice and AlfP-Cre mice is the most likely explanation for the dwarf phenotype and liver steatosis in both mouse models.

The behavioral abnormalities, reduced contextual- and cued-conditioned fear, observed in Nestin-Cre mice [11] are also likely to be caused by the expression of hGH. Several studies have shown that hGH can also activate the prolactin receptor (PRLR) in mice [20, 31]. Consistent with this is the upregulation of *Cish* that we observed. Previously, it has been shown that the expression of hGH in the endocrine pancreas also resulted in the upregulation of *Cish* [31]. However, when the mice were crossed with PRLR null mice, *Cish* was no longer upregulated. Activation of the PRLR in brain is known to be anxiolytic in both male and female rodents [34, 35]. Furthermore, a recent study has shown that male GHRH^-/-^ mice have reduced anxiety [36], indicating that activation of hypothalamic GH receptors might also be responsible for the behavioural phenotype in Nestin-Cre mice. Although the hypothalamus is important in the regulation of anxiety and stress, contextual- and cued-conditioned fear are considered to be hippocampus- and amygdala-dependent [37]. Given the broad expression of the Nestin-Cre transgene in CNS, it is well possible that receptor activation in these brain areas are responsible for, or further contribute to the behavioural phenotype.

Finally, it has been hypothesized before that the improved insulin sensitivity observed in Nestin-Cre mice could be attributed to lower GH levels [12]. However, recently it was reported that hypothalamic PRLR receptor action positively regulates hepatic insulin sensitivity [38]. In the light of this study, both mechanisms might contribute to the improved insulin sensitivity.

In conclusion, we report another artifact observed in several Cre-driver lines. In many of these strains, the hGH minigene is inserted downstream of the Cre recombinase to ensure efficient expression of the transgene. We demonstrate that this results in mouse GHD, leading to smaller mice with a metabolic and behavioural phenotype. This phenomenon has been proven for at least two different Cre lines, namely the Nestin-Cre mice, as shown in this study, and the AlfP-Cre mice [9]. Nevertheless, many more Cre mice have an insertion of the hGH minigene downstream of the Cre-recombinase. Therefore, attention should be paid when using Cre-driver lines in which the hGH minigene is used as an expression enhancer.
